# Methanol-based biosynthesis of *p*-coumaric acid by engineered *Pichia pastoris*

**DOI:** 10.1186/s40643-026-01068-7

**Published:** 2026-05-13

**Authors:** Mengyuan Chen, Jiayu Fang, Shuxian Wang, Guoxia Liu, Yanping Zhang, Yin Li, Kaizhi Jia, Taicheng Zhu

**Affiliations:** 1 Cooperative Innovation Center of Industrial Fermentation (Ministry of Education & Hubei Province), Key Laboratory of Fermentation Engineering (Ministry of Education), Hubei Key Laboratory of Industrial Microbiology, National “111” Center for Cellular Regulation and Molecular Pharmaceutics, Hubei University of Technology, Wuhan, 430068 China; 2 State Key Laboratory of Microbial Diversity and Innovative Utilization, Department of Microbial Physiological and Metabolic Engineering, Institute of Microbiology, Chinese Academy of Sciences, Beijing, 100101 People’s Republic of China; 3 University of Chinese Academy of Sciences, Beijing, 100190 People’s Republic of China

**Keywords:** *p*-Coumaric acid, Methanol, *Pichia pastoris*, Shikimate pathway, Copy number

## Abstract

*p*-Coumaric acid (p-CA) is a key aromatic precursor for the biosynthesis of flavonoids, stilbenoids, and other high-value phenylpropanoids. While microbial production of p-CA typically relies on sugar-based substrates, methanol offers a sustainable and cost-effective alternative, though its use for aromatic biosynthesis remains unexplored. Here, we report the first de novo production of p-CA from methanol using engineered methylotrophic yeast *Pichia pastoris*. Through heterologous expression of a tyrosine ammonia-lyase and implementing a balanced push–pull strategy in the shikimate pathway using feedback-resistant variants of DAHP synthase (*ARO4*) and chorismate mutase (*ARO7*), carbon flux from methanol-derived C3 and C4 precursors was effectively redirected toward aromatic biosynthesis. Shake-flask studies revealed strong gene-dosage-dependent p-CA production, but strains with high-copy numbers suffered metabolic burden under high-density fermentation. Fed-batch bioreactor cultivation demonstrated that a moderate-copy strain achieved the highest titer of 704 ± 6 mg/L, outperforming high-copy variants in robustness and scalability. This study establishes *P*. *pastoris* as a promising chassis for methanol-based aromatic production and highlights the critical trade-off between pathway amplification and cellular fitness in C1 biomanufacturing.

**Graphical abstract:**

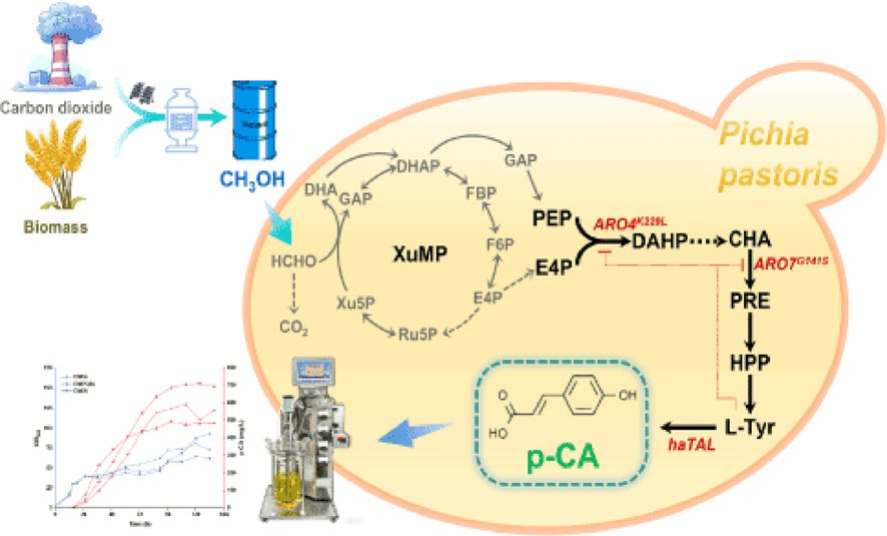

**Supplementary Information:**

The online version contains supplementary material available at 10.1186/s40643-026-01068-7.

## Introduction

*p-*Coumarate (p-CA) serves as a key precursor for a wide range of high-value compounds, including flavonoids (e.g., naringenin) (Ru et al. 2025; Wang et al. 2024a, b; Zhang et al. 2025), stilbenoids (e.g., resveratrol) (Meng et al. 2023; Zhou et al. 2025), and other polyphenolic compounds with applications in food, nutraceutical, and pharmaceutical industries (de Araújo et al. 2021; Williamson 2025). Currently, microbial production of p-CA is mainly dependent on sugar-based substrates such as glucose (Liu et al. 2019), xylose (Zhao et al. 2025), and glycerol (Kumokita et al. 2023), with titers reaching hundred-milligram to gram-per-liter scale in engineered *Escherichia coli* (Qiu et al. 2024; Zhuang et al. 2026), *Saccharomyces cerevisiae* (Liu et al. 2019; Qi et al. 2022; Zhao et al. 2025), and *Yarrowia lipolytica* (Gu et al. 2020; Zhu et al. 2024). However, the use of conventional carbohydrate feedstocks presents challenges related to cost, substrate competition, and contamination risks, driving the exploration of alternative carbon sources for sustainable bioproduction.

In recent years, methanol has emerged as an attractive feedstock for biomanufacturing due to its low cost, high purity, and potential derivation from the renewable sources such as CO_2_ and syngas (Bae et al. 2022; Wang et al. 2024a, b; Zang et al. 2025; Zhu et al. 2019). The methylotrophic yeast *Pichia pastoris* is an industrially proven host capable of efficient methanol assimilation, yet its exploitation for aromatic compound biosynthesis remains limited. Previous studies have demonstrated the capability of *P. pastoris* in generating a variety of chemicals including C2 (e.g., acetyl-CoA), C3 (e.g., pyruvate), C4 (e.g., erythrose-4-phosphate), C5 (e.g., xylulose-5-phosphate), and C6 (e.g., fructose-6-phosphate)-based chemicals (Feng et al. 2023; Guo et al. 2023; Tang et al. 2023; Wang et al. 2025a, b). However, the *de novo* biosynthesis of aromatic compounds from methanol remains unexplored, representing a significant gap in C1-based biomanufacturing.

**Fig. 1:**
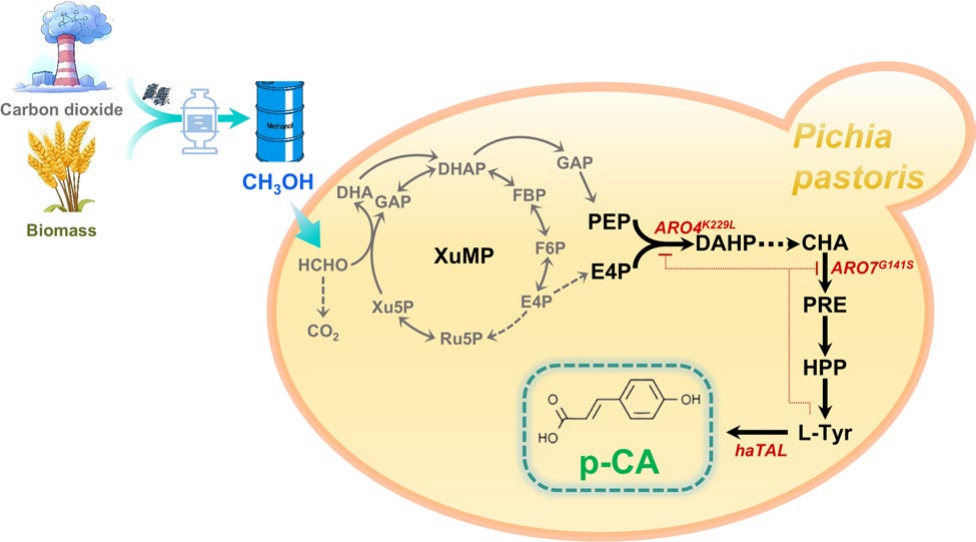
Schematic diagram of methanol-to-*p*-coumaric acid (p-CA) synthesis pathway in *P. pastoris*. Methanol is elongated through the xylulose monophosphate (XuMP) to C3 (PEP) and C4 (E4P) precursors, which then enter the shikimate pathway. The biosynthetic flux of l-tyrosine (l-Tyr) is enhanced by heterologous expression of feedback inhibition-resistant mutants *scARO4* (K229L) (encoding 3-deoxy-D-arabino-heptulosonate-7-phosphate synthase, relieving tyrosine feedback inhibition) and *scARO7* (G141S) (encoding chorismate mutase, relieving feedback inhibition). Finally, l-tyrosine is deaminated to produce the target product p-CA by heterologously expressed *haTAL* (tyrosine ammonia-lyase from *Herpetosiphon aurantiacus*). Key enzyme genes and metabolic nodes are labeled, and arrows indicate metabolic flux directions

Microbial biosynthesis of p-CA generally proceeds through the shikimate pathway, which is initiated by the condensation of phosphoenolpyruvate (PEP, C3) and erythrose-4-phosphate (E4P, C4). Thus, the availability of C3 and C4 precursors holds the key to efficient production of aromatic compounds like p-CA. In *P. pastoris*, methanol is assimilated through the xylulose monophosphate (XuMP) cycle, which natively supplies ample C3 precursors (Li et al. 2025; Zhao et al. 2024). While the availability of the C4 precursor E4P has often been a limiting factor for aromatics production in sugar-based systems (Li et al. 2023; Liu et al. 2025), our recent work demonstrated that *P. pastoris* grown on methanol can generate substantial E4P flux, sufficient to drive high-level production of erythritol (Wang et al. 2025a, b). This indicates that the native methanol metabolism in *P*. *pastoris* grown can potentially supply the necessary C3 and C4 building blocks for an efficient shikimate pathway, as conceptually illustrated in Fig. 1.

In this study, we report the first de novo biosynthesis of *p*-CA from methanol using engineered *P. pastoris*. We constructed the pathway by introducing a heterologous tyrosine ammonia-lyase and implemented a balanced “push-pull” strategy within the shikimate pathway using feedback-resistant variants of key enzymes, DAHP synthase and chorismate mutase, to enhance carbon flux from methanol towards p-CA. Beyond pathway engineering, we also examined the critical trade-off between pathway amplification (via multi-copy integration) and cellular fitness during scale-up from flasks to bioreactors. Our work not only establishes *P. pastoris* as a promising platform for methanol-based aromatic production but also provides key insights into balancing metabolic burden and productivity in C1 fermentation processes, which is essential for sustainable industrial biomanufacturing.

## Materials and methods

### Strains, plasmids, and cultivation conditions

*Escherichia coli* DH5α (Tiangen Biotech, China) was used for plasmid construction and propagation. *P*. *pastoris* GS115 or Δ*Ku70* served as the parental strain for *p*-coumaric acid (p-CA) production. All strains used in this study are listed in Table S1. pMPICZ and pL3Z-His were used for single- and multiple-gene overexpression. The BB3cN_pGAP_23*_pPFK300_Cas9 plasmid (Gassler et al. 2019) was used for CRISPR–Cas9-based gene integration. Detailed plasmid information is summarized in Table S2.

*E. coli* was cultivated in LB medium at 37 °C and 220 rpm. *P*. *pastoris* strains were routinely cultivated in YPD medium at 30 °C and 220 rpm, with agar added at 20 g/L for solid medium. Zeocin (100 mg/L) or Nourseothricin (200 mg/L) was added when required.

### Genetic manipulation and strain construction

The p-CA biosynthetic pathway was constructed using three heterologous genes: *scARO4* (K229L), encoding a feedback inhibition–resistant 3-deoxy-D-arabino-heptulosonate-7-phosphate synthase from *S. cerevisiae*; *scARO7* (G141S), encoding a feedback-resistant chorismate mutase from *S. cerevisiae*; and *haTAL*, encoding tyrosine ammonia-lyase from *Herpetosiphon aurantiacus*. All genes were codon-optimized for expression in *P. pastoris* and synthesized by GenScript (Nanjing, China).

Single-gene expression plasmids were constructed by cloning *scARO4*^*fbr*^, *scARO7*^*fbr*^, and *haTAL* into pMPICZ using Gibson Assembly, yielding pMPICZ-*scARO4*^*fbr*^, pMPICZ-*scARO7*^*fbr*^, and pMPICZ-*haTAL*, respectively. Dual- and tri-gene expression plasmids were assembled into pL3Z-His via *BsaI* sites using Golden Gate assembly.

For CRISPR-mediated integration, a guide RNA targeting the NS1 locus was designed and cloned into BB3cN_pGAP_23*_pPFK300_Cas9. The resulting plasmid was co-transformed with the assembled tri-gene cassette (*scARO4*, *scARO7*, and *haTAL*) flanked by NS1 homologous arms. For single-crossover knock-in, pL3Z-His-based plasmids containing dual or triple shikimate pathway genes were linearized with *Sal*I and introduced into competent *P*. *pastoris* cells by electroporation. Positive transformants were verified by colony PCR and are summarized in Table S1.

### Whole-genome sequencing and foreign gene copy number analysis

To investigate differences in p-CA production among selected strains, whole-genome sequencing was performed. Next generation sequencing library preparations were constructed following the manufacturer’s protocol. For each sample, 200 µg genomic DNA was randomly fragmented by Covaris to an average size of 300–350 bp. The fragments were treated with End Prep Enzyme Mix for end repairing, 5’ Phosphorylation and 3’ adenylated, to add adaptors to both ends. Size selection of Adaptor-ligated DNA was then performed by DNA Cleanup beads. Each sample was then amplified by PCR for 8 cycles using P5 and P7 primers, with both primers carrying sequences which can anneal with flowcell to perform bridge PCR and P7 primer carrying a six-base index allowing for multiplexing. The PCR products were cleaned up and validated using an Agilent 2100 Bioanalyzer. The qualified libraries were sequenced pair end PE150 on the illumina HiseqXten/Novaseq/MGI2000 System.

After quality filtering, clean reads were aligned to the *P*. *pastoris* reference genome. Genome coverage and sequencing depth were calculated, and the sequencing depth of heterologous genes (*scARO4*, *scARO7*, and *haTAL*) was extracted to infer relative copy number differences among strains.

### Quantitative PCR analysis of foreign gene copy number

Genomic DNA was extracted using a commercial yeast genomic DNA kit. Quantitative real-time PCR was performed using genomic DNA as the template with FastFire qPCR PreMix with gene-specific primers listed in Table S3. The *P*. *pastoris GAPDH* gene was used as the internal reference. Relative gene copy numbers were calculated using the 2^−ΔΔCt^ method based on three biological replicates, each with three technical replicates (Zhu et al. 2009a, b).

### Shake-flask fermentation

Single colonies were pre-cultured in YPD (10 g/L yeast extract, 20 g/L tryptone, 20 g/L glucose) medium and used as seed cultures. Cells were harvested and resuspended in BSM (Wang et al. 2025a, b) medium (with or without 2% yeast extract) or Buffered Methanol-complex Medium (BMMY) medium (Miao et al. 2021) in 250 mL baffled flasks with a working volume of 25 mL. Fermentation was initiated at an OD_600_ of 4.0 and conducted at 30 °C and 220 rpm for 96 h. Methanol was used as the sole carbon source and supplemented every 24 h to maintain a concentration of 1%–2% (v/v). Samples were collected at defined time points for biomass and p-CA analysis.

### Fed-batch fermentation in bioreactor

Fed-batch fermentation was carried out in a 1 L bioreactor containing 0.8 L BSM supplemented with 2% yeast extract. Seed cultures were inoculated at 5% (v/v). Temperature and pH were maintained at 30 °C and 6.0, respectively. Dissolved oxygen (DO) was manually maintained at approximately 20% by adjusting agitation and aeration throughout the entire fermentation.

The fermentation process consisted of a glycerol batch phase, a methanol adaptation phase, and a methanol feeding phase for p-CA production. Methanol feeding was automatically controlled to maintain a concentration of 3 g/L.

### Analytical methods

Biomass concentration was estimated by measuring OD_600_ after appropriate dilution. p-CA was extracted from fermentation broth as follows: 200 µL of fermentation broth was mixed with 800 µL of methanol, thoroughly vortexed for 10 min, and centrifuged at 12,000 rpm for 5 min to collect the supernatant. p-CA concentration was determined by high-performance liquid chromatography (HPLC) using a C18 column with a phosphoric acid–acetonitrile gradient and UV detection at 280 nm. Quantification was performed using an external standard curve generated with authentic p-CA standards.

## Results

### Construction of a *p*-coumaric acid biosynthetic pathway from methanol in *P. pastoris* and its impact on yeast physiology

**Fig. 2:**
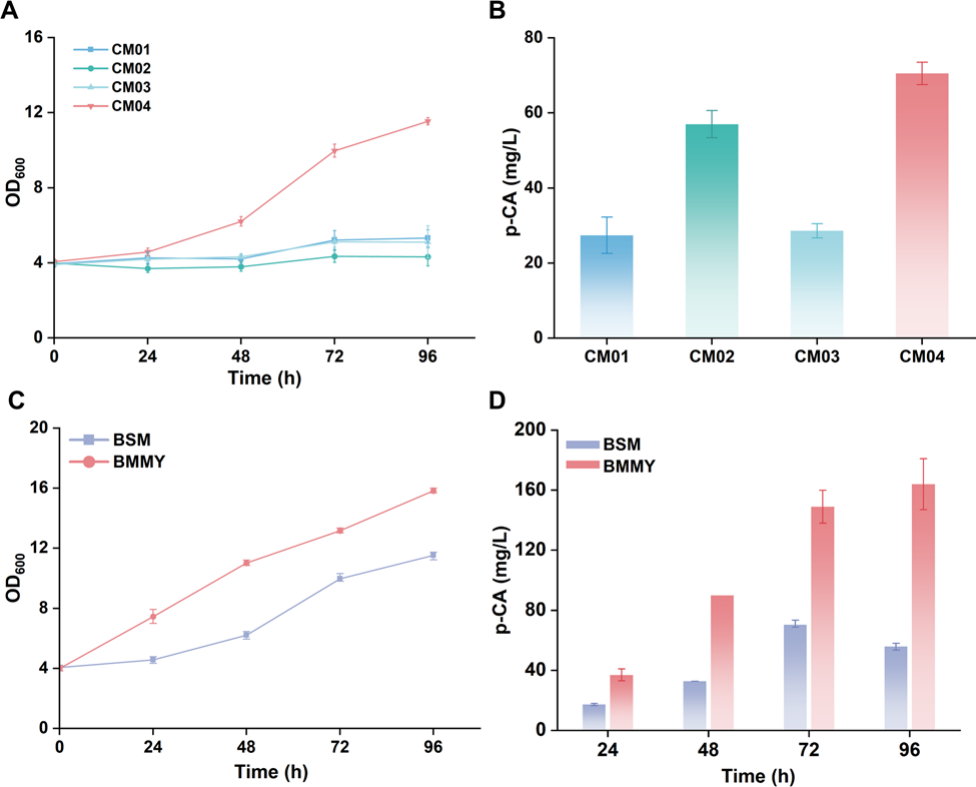
*p*-Coumaric acid production and growth profiles of different engineered strains in shake-flask fermentations. **A** Growth curves (OD_600_) of strains CM01 (*haTAL*), CM02 (*haTAL*, *ARO4*^*fbr*^), CM03 (*haTAL*, *ARO7*^*fbr*^), and CM04 (*haTAL*, *ARO4*^*fbr*^, *ARO7*^*fbr*^) in Basal Salt Medium (BSM). **B** Corresponding p-CA titers achieved by the strains after 96 h of fermentation in BSM. **C** Comparison of growth and p-CA production for strain CM04 in complex medium (BMMY) versus defined BSM medium. **D** Time-course p-CA titers of strain CM04 in BSM and BMMY media from 24 to 96 h. Data points represent mean ± standard deviation (s.d.) of three biological replicates (*n* = 3)

p-CA can be biologically produced through the deamination of l-tyrosine, catalyzed by tyrosine ammonia-lyase (*TAL*, EC 4.3.1.23) (Fig. 1). Since *P*. *pastoris* natively lacks this enzyme, a codon-optimized *TAL* gene from *Herpetosiphon aurantiacus* (*haTAL*) was expressed under the control of the strong *AOX1* promoter and integrated into the *P*. *pastoris* genome, resulting in the starting strain CM01. During shake-flask fermentation, the cells were first cultured in YPD medium to boost biomass accumulation before being harvested and resuspended in BSM medium with methanol for induction (initial OD_600_ = 4). Strain CM01 achieved a p-CA titer of 27 ± 5 mg/L (Fig. 2B), demonstrating the successful construction of the complete p-CA biosynthetic pathway from methanol.

To maximize C1 carbon flux toward p-CA, we next alleviated the key regulatory bottlenecks of the native shikimate pathway. DAHP synthase (encoded by *ARO4*) and chorismate mutase (encoded by *ARO7*) are subject to feedback inhibition by aromatic amino acids like l-tyrosine and thus the major targets for engineering (Liu et al. 2019). To relieve this negative regulation, feedback-inhibition-resistant mutants *scARO4* (K229L) (*ARO4*^*fbr*^) and *scARO7* (G141S) (*ARO7*^*fbr*^) were first overexpressed individually in the CM01 background, yielding strains CM02 and CM03, respectively. For construction convenience and to avoid the need for an additional selection marker, CM04, co-expressing *haTAL*, *ARO4*^*fbr*^, and *ARO7*^*fbr*^, was generated by CRISPR-Cas9-mediated integration of an in vitro assembled three-gene cassette. To exclude possible gene-dosage effects, the copy numbers of the integrated genes in CM01, CM02, and CM03 were determined by qPCR, which confirmed that each strain carried a single copy of its corresponding integrated gene cassette. Fermentation results showed that overexpressing *ARO7*^*fbr*^ alone in CM03 did not significantly improve the p-CA titer, whereas solely overexpressing *ARO4*^*fbr*^ (CM02) increased the titer to 57 ± 4 mg/L. Combined overexpression of *ARO4*^*fbr*^ and *ARO7*^*fbr*^ in CM04 further enhanced the titer to 70 ± 3 mg/L. These results indicate that both “push” of the C3/C4 precursors into the shikimate pathway (via *overexpressing ARO4*^*fbr*^) and downstream “pull” of metabolic intermediates (via overexpressing *ARO7*^*fbr*^*)* are essential for improving p-CA production from methanol.

In addition to titers, the growth profiles of the engineered strains were also investigated in this study. The results revealed that CM01 exhibited pronounced growth inhibition in BSM medium, showing only minimal biomass increase after induction, which is likely due to depletion of the essential amino acid l-tyrosine caused by p-CA synthesis. Interestingly, enhancing upstream flux via *ARO4*^*fbr*^ overexpression in CM02 further aggravated growth inhibition, probably because increased flux towards shikimate pathway promotes the accumulation of inhibitory intermediates such as *p*-hydroxyphenylpyruvate (Kuivanen et al. 2021). Consistently, co-expression of *ARO7*^*fbr*^ with *ARO4*^*fbr*^ in CM04 largely alleviated the host growth defect, suggesting that boosting chorismate mutase facilitates downstream channeling of pathway intermediates and reduces their inhibitory effects. Collectively, these results highlight the importance of balancing pathway “push” (*ARO4*) and “pull” (*ARO7*) to improve cellular fitness as well as p-CA production.

To further relieve the metabolic burden associated with p-CA synthesis, we replaced the chemically defined BSM medium with the complex medium BMMY. The results showed that the cell growth of CM04 was significantly improved, reaching a final OD_600_ of 15.8, as opposed to approximately 10 in BSM medium. Consequently, the p-CA titer increased substantially, reaching 164 ± 17 mg/L (Fig. 2C, D).

### Generation of high p-CA-producing strains via single-crossover integration

Following the identification of the three key genes (*scARO4*^*fbr*^, *scARO7*^*fbr*^, *haTAL*) required for efficient p-CA synthesis, we evaluated the impact of different strain construction strategies, including host strain background and genomic integration method. The three gene cassettes were assembled into the pL3Z expression vector via Golden Gate Assembly, thus generating plasmid pL3Z-ARO47TAL (Suppl. Table S1). This vector contains a Zeocin resistance marker and the *HIS4* sequence for single-crossover integration genomic integration. The plasmid was linearized and transformed into the GS115 and the Δ*Ku70* strain, respectively.

To assess clonal consistency, transformants from both backgrounds were selected and evaluated for p-CA production. The results indicated that GS115-based transformants exhibited uniform production levels among the three tested clones; consequently, a representative clone was selected and designated as CM06. In contrast, transformants derived from the Δ*Ku70* background displayed significant variation in p-CA titers. From this population, two distinct clones exhibiting divergent production capabilities in a preliminary screening were selected for further characterization: the higher-producing clone CM05a and the lower-producing clone CM05b.

**Fig. 3:**
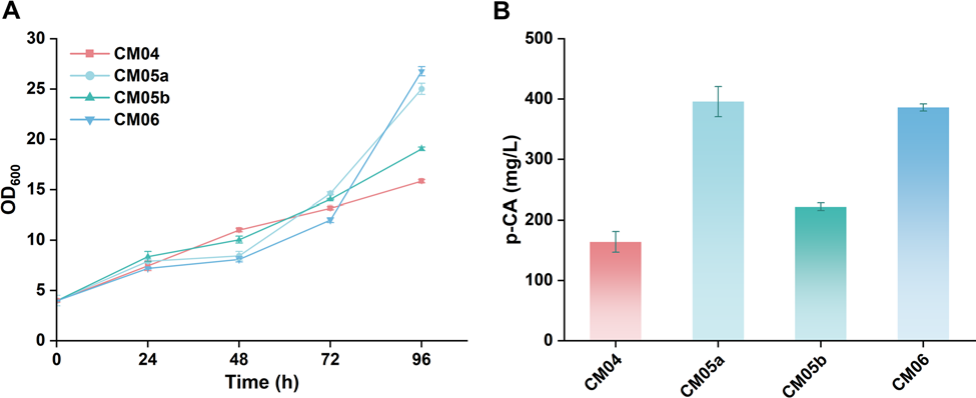
Evaluation of *p-*coumaric acid production across different host backgrounds and clones **A** Time-course profiles of p-CA production during shake-flask fermentation in BMMY medium for strains CM04, CM05a, CM05b, and CM06. **B** Final p-CA titers at 96 h for the corresponding strains. Data are presented as mean ± s.d. (*n* = 3)

These newly constructed strains (CM05a, CM05b, and CM06) were subsequently evaluated in shake flasks using BMMY complex medium, with the previously constructed strain CM04 serving as a control. All three strains exhibited significantly higher p-CA production compared to CM04 (Fig. 2C). Specifically, strains CM05a and CM06 achieved titers of 396 ± 25 mg/L and 386 ± 6 mg/L at 96 h, respectively. The lower-producing clone CM05b achieved a titer of 222 ± 6 mg/L at 96 h, which was still higher than the control strain CM04 (164 ± 17 mg/L) (Fig. 3A, B). These results demonstrate that both the host strain and integration strategy has a significant impact on production performance. Notably, the single-crossover integration strategy yielded clones with remarkably higher p-CA titers than the CRISPR-Cas9-mediated integration strategy.

### Genetic basis of p-CA production variation among engineered strains revealed by whole-genome sequencing (WGS) and qPCR

To elucidate the genetic basis for the p-CA production variation observed among the engineered strains constructed with different strategies, we performed a comparative genomic analysis using whole-genome sequencing (WGS). We sequenced three representative clones: the high-producing strain CM05a, the moderate-producing strain CM05b, and the low-producing control CM04.

**Fig. 4:**
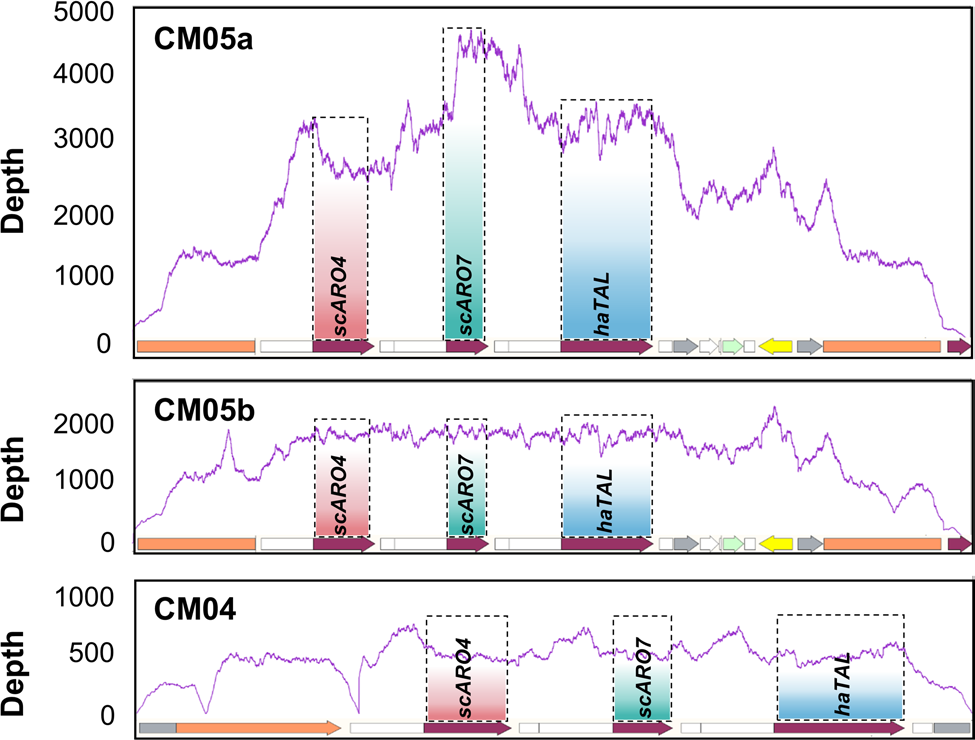
Comparative analysis of sequencing depth across heterologous gene regions in different engineered strains. Whole-genome sequencing was used to analyze the sequencing depth at the integration loci of the heterologous genes *scARO4* (K229L), *scARO7* (G141S), and *haTAL* in the high-producing strain CM05a, the moderate-producing strain CM05b, and the low-producing strain CM04

Comparative analysis against the *P*. *pastoris* GS115 reference genome revealed that, while all the sequenced strains exhibited structurally intact genomes with no significant chromosomal aberrations, pronounced differences in sequencing depth were observed across the heterologous p-CA pathway genes (Fig. 4). In the high-producing strain CM05a, the sequencing depth over the integrated *scARO4* (K229L), *scARO7* (G141S), and *haTAL* loci was markedly elevated, ranging from approximately 2500 × to 4000 ×, with consistently high coverage across the three gene regions. In contrast, the moderate-producing strain CM05b showed a lower but still enriched depth of ~ 1500 ± 100 × across the same loci, while CM04 exhibited the lowest coverage, at only ~ 500 × – 600 ×. The concordant depth shifts across *ARO4*^*fbr*^, *ARO7*^*fbr*^, and *haTAL* within each strain indicate that the gene dosage of the introduced modules differs substantially among selected strains.

**Fig. 5:**
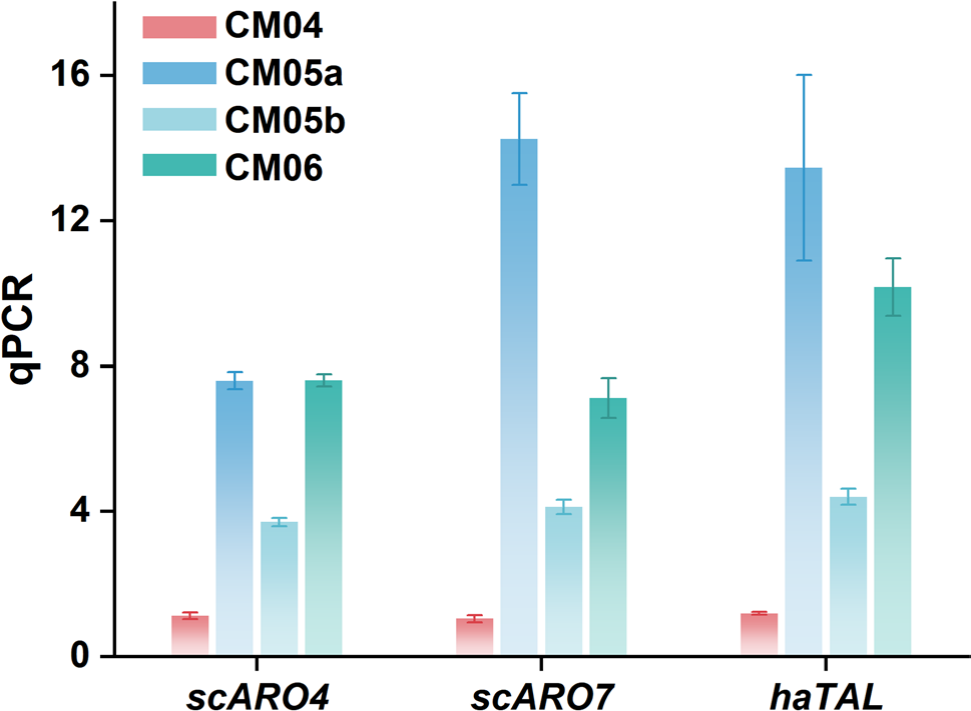
Quantification of heterologous gene copy numbers in engineered strains by qPCR. The relative copy numbers of *scARO4* (K229L), *scARO7* (G141S), and *haTAL* in strains CM04, CM05a, CM05b, and CM06 were determined by quantitative real-time PCR (qPCR), normalized to the endogenous *GAPDH* gene. Data are expressed as mean ± s.d. from three biological replicates (*n* = 3)

To further validate these findings, we determined the copy numbers of *scARO4*, *scARO7*, and *haTAL* in strain CM04, CM05a and CM05b, along with another high-producing strain CM06, via qPCR (Fig. 5). The analysis revealed a strong positive correlation between total gene dosage and p-CA titer. The high-producing strains (CM06 and CM05a) were characterized by a high gene dosage, carrying a total of ≥ 25 copies of the pathway genes (e.g., CM05a: 8 *ARO4*, 14 *ARO7*, 13 *haTAL*). The moderate-producing strain CM05b carried four copies of each pathway gene (Total: 12). As expected, the low-producing control CM04 harbored only a single copy of each pathway gene.

These findings demonstrate that p-CA hyper-production is primarily driven by high exogenous gene dosage. Furthermore, the single-crossover integration strategy consistently generated multi-copy integrants—likely driven by the selective pressure of high Zeocin concentrations—whereas the CRISPR-Cas9 method favored single-copy integration.

### Optimization of fermentation medium to support methanol-based *p*-coumaric acid production

**Fig. 6:**
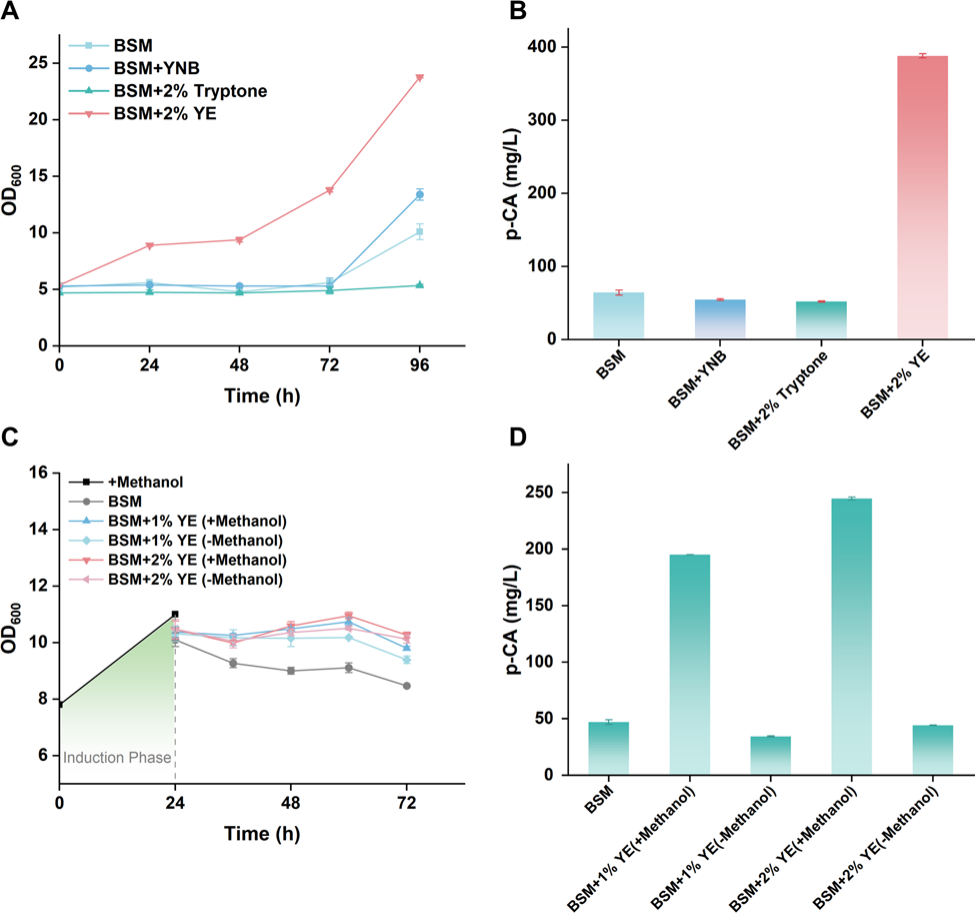
Optimization of fermentation medium to mitigate the metabolic burden associated with p-CA synthesis. **A**, **B** Effects of supplementing the defined BSM medium with 2% Tryptone, 0.5% Yeast Nitrogen Base without amino acids (YNB), or 2% Yeast Extract (YE) on the growth (**A**) and p-CA production (**B**) of strain CM04. **C**, **D** Comparison of growth (**C**) and p-CA production (**D**) for CM04 in BSM + 2% YE with or without methanol. Data points represent mean ± s.d. (*n* = 3)

The p-CA–producing strains exhibited severe growth inhibition when cultivated in chemically defined BSM medium; however, this phenotype was alleviated when cells were grown in complex BMMY medium. To identify the nutritional components in BMMY responsible for supporting cell fitness and p-CA production, we performed a component supplementation assay using BSM as the basal medium. BSM was individually supplemented with the key constituents of the complex BMMY medium: 2% tryptone, 0.5% yeast nitrogen base without amino acids (YNB), or 2% yeast extract (YE) (Fig. 6A, B).

The results demonstrated that supplementation with 2% YE was sufficient to restore cell growth and production, achieving a final OD_600_ of 27 ± 1 and a p-CA titer of 392 ± 11 mg/L at 96 h. In contrast, supplementation with tryptone or YNB failed to improve strain performance, yielding markedly lower biomass (OD_600_: 12 ± 1 and 10 ± 1, respectively) and titers (< 110 mg/L). This indicates that YE provides essential nutrients required to support the metabolic demand of the high-copy strain.

Since yeast extract is rich in free amino acids, including l-Tyr, the direct precursor for p-CA synthesis, it was necessary to determine whether the increased p-CA titer upon YE supplementation was driven by improved overall cell fitness or simply by the bioconversion of the exogenous precursors present in YE. To this end, a biotransformation experiment was performed in which methanol-induced yeast cells were harvested and resuspended in BSM supplemented with 1% or 2% YE, either with or without methanol, and p-CA titers under the different conditions were compared (Fig. 6C, D).

In the absence of methanol, the strain produced only 39 ± 4 mg/L of p-CA at 72 h, representing merely 18% of the titer achieved in the presence of methanol (212 ± 10 mg/L). This result suggests that the main function of YE is to supply essential growth factors (e.g., vitamins and trace nutrients) necessary for biomass accumulation, rather than providing direct substrates for bioconversion. Accordingly, BSM supplemented with 2% YE was selected as the optimal medium for subsequent scale-up in bioreactors.

### Evaluation of *p*-coumaric acid production in fed-batch bioreactor fermentation

To assess the industrial potential of the engineered strains and investigate the impact of gene dosage on process scalability, we performed high-density fed-batch fermentation in a 1-L bioreactor. Three representative strains with varying p-CA biosynthetic gene dosages were evaluated: CM04 (single copy, low dosage), CM05b (multi-copy, moderate dosage), and CM06 (multi-copy, high dosage). Based on our medium optimization results, a modified BSM medium supplemented with 20 g/L yeast extract was employed. The fermentation process comprised three phases: a glycerol batch phase (0–12 h) for biomass accumulation, a methanol adaptation phase (12–15 h) to induce the *AOX1* promoter, and a production phase (15–108 h) during which the methanol concentration was maintained at 3–8 g/L.

**Fig. 7:**
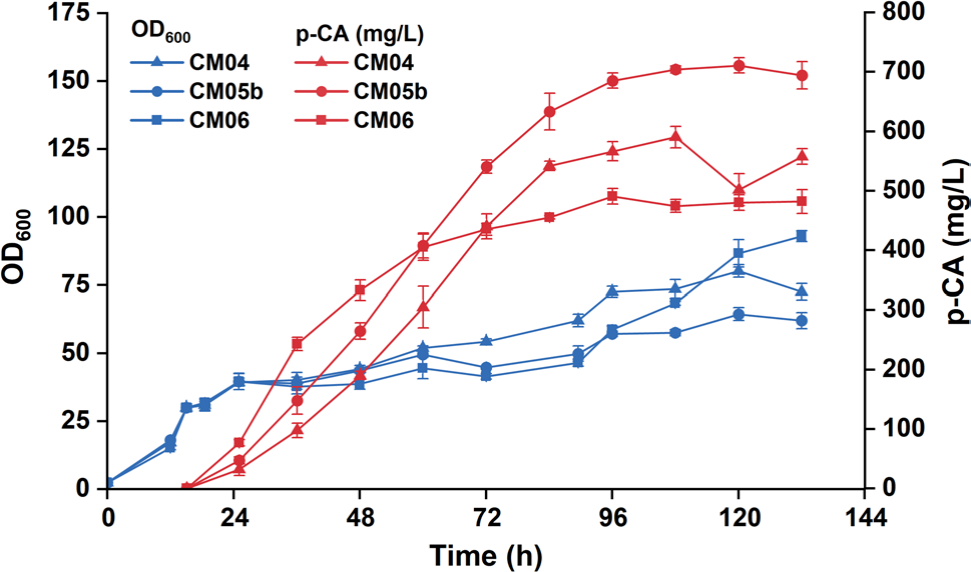
Fermentation profiles of engineered strains with low-to-high copy numbers of heterologous pathway genes during fed-batch fermentation using methanol as the carbon source in a 1-L fermentor

The fermentation results revealed a striking reversal in strain performance compared to the shake-flask experiments (Fig. 7). In the shake-flask scale, the high-copy strain CM06 had demonstrated the highest production. However, under high-density bioreactor conditions, CM06 exhibited the lowest performance, achieving a final p-CA titer of 474 ± 11 mg/L after 96 h of induction. Notably, during the late production phase, CM06 showed strong signs of physiological stress, including a gradual rise in pH and observed cell death by methylene blue stain (data not shown), suggesting that the heavy metabolic burden imposed by the high gene dosage compromised cell viability during prolonged high-density culture.

In contrast, the strains with lower gene dosages demonstrated significantly better scalability. The single-copy strain CM04, which performed poorly in shake flasks, achieved a titer of 590 ± 18 mg/L in the bioreactor, representing a 3.6-fold increase relative to its flask titer. Notably, the moderate-copy strain CM05b emerged as the most robust candidate, achieving the highest titer of 704 ± 6 mg/L—a 3.17-fold increase over its flask performance.

These findings highlight a critical trade-off between pathway flux and cellular fitness. While maximizing gene dosage (as in CM06) is beneficial in shake-flask conditions, it imposes an excessive metabolic load that becomes detrimental under the fermentor-scale fermentation. The superior performance of CM05b indicates that an optimal, moderate gene dosage is essential for balancing biosynthetic capability with physiological robustness, thereby maximizing final product titers in fed-batch processes.

## Discussion

*p-*Coumaric acid (p-CA) is an important aromatic compound and the precursor for a variety of useful plant-derived compounds (Kumokita et al. 2022; Ru et al. 2025). Most reported microbial p-CA production systems rely on sugar-based substrates, such as glucose, glycerol, or xylose (Kumokita et al. 2023; Liu et al. 2019; Zhao et al. 2025). In this work, we demonstrate the first successful methanol-based production of the aromatic compound *p-*coumaric acid in the methylotrophic yeast *P*. *pastoris*, achieving a titer of 704 ± 6 mg/L in fed-batch bioreactor fermentation, although this remains lower than those reported for glucose-based platforms (1.5–12.5 g/L in E. coli or S. cerevisiae) (Jeong et al. 2025; Liu et al. 2019). The yield obtained in this study (3.6 mg/g methanol) was also lower than those reported for glucose-based systems (20.0-154.9 mg/g) (Liu et al. 2019; Qiu et al. 2024; Zhuang et al. 2026), likely due in part to carbon loss through methanol dissimilation and methanol evaporation during fermentation. Based on stoichiometric analysis of the XuMP pathway, glycolysis, and the shikimate pathway, the biosynthesis of one mole of p-CA from methanol generally requires a minimum of 11 mol of methanol, although the values may vary depending on the metabolic routes. Of these, 10 mol are required to provide the carbon skeletons for the precursors PEP and E4P, and 1 additional mol is needed to generate reducing power. This corresponds to a theoretical maximum yield of approximately 0.47 g/g. Therefore, as commonly observed in microbial p-CA production, the experimental yield achieved thus far remained substantially below the theoretical maximum, highlighting the difficulty of redirecting sufficient central carbon flux into the shikimate pathway. Despite these limitations, this work establishes methanol as a feasible feedstock for aromatic compound biosynthesis and represents an important step toward sustainable C1-based biomanufacturing.

The study shows that the introduction of the heterologous *TAL* is sufficient for the completeness of p-CA synthesis from methanol to C4 and C3 building blocks and finally to p-CA. However, to further enhance p-CA production, as reported for sugar-based systems (Liu et al. 2019; Qiu et al. 2024), a “push and pull” strategy applies. The overexpression of the feedback-inhibition-resistant mutant of *ARO4* is the crucial tuning point for pushing the C4 and C3 flux towards the shikimate pathway. Without *ARO4*^*fbr*^ overexpression, overexpression of downstream control nodes did not increase the product titer. After introducing *ARO4*^*fbr*^, the downstream “pull” through overexpression of *ARO7*^*fbr*^ is another important strategy. Results showed that overexpression of *ARO7*^*fbr*^ on top of *ARO4*^*fbr*^ not only further improved p-CA production but also relieved cell growth inhibition, suggesting that this balanced push–pull regulation likely facilitates more efficient channeling of intermediates through the shikimate pathway.

Moreover, we observed a remarkable gene dosage effect for pathway amplification and p-CA production. Whole-genome sequencing and qPCR analysis revealed that, compared with CRISPR-mediated integration, single-vector integration generated clones with varying copy numbers, including high-copy strains. The results showed a positive correlation between the copy number of the three genes and the p-CA titers in shake flask culture and also at the initial stage of fermentor culture. This observation is consistent with previous reports in methylotrophic systems, where strong promoters combined with multi-copy integration are often required to achieve high pathway throughput (Jiang et al. 2024).

In addition to p-CA synthesis, the effects of p-CA synthesis on the physiology of yeast cells are also noticeable and have an important impact on the products. The engineering of the p-CA synthesis pathway seems to significantly inhibit cell growth during methanol culture. This inhibition seems to come from multiple levels. This could be due to the depletion of the intracellular aromatic pool size (e.g. l-Tyr), as overexpression of the *TAL* gene alone in *P*. *pastoris* could severely inhibit cell growth in chemically defined medium. Also, the shikimate intermediates seem toxic to yeast cells, as reported by previous studies (Kumokita et al. 2022). We found overexpression of *ARO4*^*fbr*^ without *ARO7*^*fbr*^ would lead to more severe growth inhibition, while combining it with *ARO7*^*fbr*^ would remarkably restore cell growth. To mitigate this, we found the use of complex medium—in particular, the addition of yeast extract—would support cellular fitness, perhaps by supplying essential growth factors or energy. Further work is under investigation to dissect the role of yeast extract in promoting cell growth and p-CA production.

Furthermore, while high-copy strains outperformed lower-copy variants in shake-flask cultures, their advantage diminished—and ultimately reversed—under high-density fermentor conditions. This failure in scale-up was likely because the inhibitory effect was more pronounced in fermentor culture, perhaps due to the more fully induced *AOX1* promoter under fermentor conditions (Cámara et al. 2017; Qian et al. 2021). The great loss of viability was evidenced by the increased pH and stained cells under microscope observation.

To assess whether methanol-related toxicity contributed to the poor performance of the high-copy strain CM06 during fermentor cultivation, we calculated the specific methanol consumption rate (q_s_) during the first 45 h of fed-batch fermentation. Notably, CM06 exhibited the lowest q_s_ [0.14 g/(g·h)] among the three strains, compared with 0.22 g/(g·h) for CM04 and 0.19 g/(g·h) for CM05b. This observation suggests that the reduced viability of CM06 was unlikely to result from an excessively high methanol oxidation rate and the associated accumulation of toxic intermediates such as formaldehyde. Rather, this phenotype was more likely attributable to the excessive metabolic burden imposed by the high copy number of the heterologous pathway genes, including increased precursor drain, pathway imbalance, and the accumulation of inhibitory intermediates, as discussed above. Together, these results highlight the importance of balancing between pathway strength and host physiology in methanol-based production systems.

The study also points to two additional considerations for future strain optimization. First, during fed-batch fermentation of all three strains, HPLC analysis detected a peak corresponding to shikimate, which was confirmed by mass spectrometry as the major component (data not shown). This result suggests that downstream steps of the shikimate pathway may constitute a major bottleneck in p-CA biosynthesis and thus represent important targets for future engineering.

Second, genetic stability is also an important factor to consider in the further development of this methanol-based production system. Previous studies on methanol-induced protein expression in multi-copy strains have shown that strains with medium to high copy numbers may undergo significant gene loss after methanol induction, likely due to recombination-mediated loop-out of highly repeated gene modules (Zhu et al. 2009a, b). Therefore, although single-crossover integration enables rapid construction of multi-copy strains for proof-of-concept studies, its long-term stability may require careful evaluation. In contrast, CRISPR-Cas9-mediated integration offers better control over integration locus and copy number and may provide improved genetic stability. For future strain engineering, introducing multiple copies at distinct genomic loci using CRISPR-Cas9 may therefore represent a preferable strategy.

In conclusion, this study demonstrates the feasibility of producing *p*-coumaric acid from methanol in the methylotrophic yeast *P*. *pastoris*. By implementing a balanced push–pull strategy around the shikimate pathway, carbon flux toward aromatic biosynthesis was effectively enhanced while maintaining cellular fitness. We further show that gene dosage is a key driver of production but must be carefully tuned to avoid excessive metabolic burden, particularly under high-density fermentation conditions. These findings provide practical design principles for methanol-based production of aromatic fine chemicals in methylotrophic hosts.

## Supplementary Information

Below is the link to the electronic supplementary material.

Supplementary Material 1

## Data Availability

All data generated or analysed during this study are included in this published article and its supplementary information files. Further datasets from the current study can be obtained from the corresponding authors upon reasonable request.
