# Supplementary material for: Methanol-based biosynthesis of p-coumaric acid by engineered Pichia pastoris
Source: Bioresour Bioprocess. 2026 May 13;13(1):68. doi: 10.1186/s40643-026-01068-7 (PMC13172077; doi:10.1186/s40643-026-01068-7)
Supplement: Supplementary file 1 — Supplementary Material 1 [file 40643_2026_1068_MOESM1_ESM.docx]

**Supporting Information**

**Methanol-based biosynthesis of *p*-coumaric acid in the methylotrophic yeast *Pichia pastoris* (*Komagataella phaffii*)**

**AUTHOR**

Mengyuan Chen^1,2^, Jiayu Fang^2,3^, Shuxian Wang^2,3^, Guoxia Liu^2^, Yanping Zhang^2^, Yin Li^2^, Kaizhi Jia^1,*^, Taicheng Zhu^2,3,*^

**AFFILIATION**

^1^ Cooperative Innovation Center of Industrial Fermentation (Ministry of Education & Hubei Province), Key Laboratory of Fermentation Engineering (Ministry of Education), Hubei Key Laboratory of Industrial Microbiology, National “111” Center for Cellular Regulation and Molecular Pharmaceutics, Hubei University of Technology, Wuhan 430068, China

^2^ CAS Key Laboratory of Microbial Physiological and Metabolic Engineering, State Key Laboratory of Microbial Resources, Institute of Microbiology, Chinese Academy of Sciences, Beijing 100101, P.R. China

^3^ University of Chinese Academy of Sciences, Beijing 100190, People’s Republic of China

*Corresponding author. E-mail:[kaizhijia@hbut.edu.cn](mailto:kaizhijia@hbut.edu.cn)

**Supplementary Tables**

**Table S1. Strains constructed in this study.**

| **Strains** | **Description** | **Source** |
| --- | --- | --- |
| *E. coli* DH5α | Commercial transformation host for cloning | Invitrogen |
| *E.coli* trans2-Blue | Commercial transformation host for cloning | Invitrogen |
| *P. pastoris* GS115 | Commercial transformation host for  Cloning; *his4*^-^, Mut^+^ | Laboratory stock |
| CM00 | GS115, Δ*KU70*; *HIS4*+ | Laboratory stock |
| CM01 | CM00 harboring linearized vector pMPICZ-*haTAL* via electroporation | This study |
| CM02 | CM00 harboring linearized vector pL3Z-ARO4TAL via electroporation | This study |
| CM03 | CM00 harboring linearized vector pL3Z-ARO7TAL via electroporation | This study |
| CM04 | CM00 with pL3Z-ARO47TAL integrated into int1 locus via CRISPR-Cas9-mediated homologous recombination | This study |
| CM05 | CM00 harboring linearized vector pL3Z-ARO47TAL via electroporation | This study |
| CM06 | P. pastoris GS115 harboring linearized vector pL3Z-ARO47TAL via electroporation | This study |

**Table S2. Plasmids constructed in this study.**

| **plasmids** | **Description** | **Source** |
| --- | --- | --- |
| pPICZA | Vector for extracellular expression recombinant protein carrying Zeo^R^ | Laboratory stock |
| pL3Z-*His* | Shuttle vector carrying the *HIS4* gene (for single-crossover genomic integration) and Zeocin resistance marker; used for single- and multiple-gene overexpression in P. pastoris | Laboratory stock |
| BB3cN_pGAP_23*_pPFK300_Cas9 | CRISPR-Cas9-based gene integration plasmid with GAP promoter-driven Cas9 expression, harbors a nourseothricin resistance gene; used for targeted genomic integration (Gassler et al. 2019) | Commercial purchase (Gassler et al. 2019) |
| K-PGAP-Cas9-gRNA-int1 | CRISPR-Cas9 vector designed for foreign gene integration into the int1 locus of P. pastoris, containing a GAP promoter-driven Cas9 gene and a guide RNA (gRNA) targeting the int1 site | This study |
| pMPICZ-*haTAL* | pPICZ A-derived vector harboring the haTAL gene (codon-optimized for P. pastoris; encodes tyrosine ammonia-lyase from Herpetosiphon aurantiacus) | This study |
| pMPICZ-*ARO4* | pPICZ A-derived vector harboring the scARO4 (K229L) gene (codon-optimized for P. pastoris; encodes a feedback-resistant 3-deoxy-D-arabino-heptulosonate-7-phosphate synthase from Saccharomyces cerevisiae) | This study |
| pMPICZ-*ARO7* | pPICZ A-derived vector harboring the scARO7 (G141S) gene (codon-optimized for P. pastoris; encodes a feedback-resistant chorismate mutase from Saccharomyces cerevisiae) | This study |
| pL3Z-ARO4TAL | pL3Z-His-derived vector assembled via Golden Gate cloning, harboring scARO4 (K229L) and haTAL genes for co-expression in P. pastoris | This study |
| pL3Z-ARO7TAL | pL3Z-His-derived vector assembled via Golden Gate cloning, harboring scARO7 (G141S) and haTAL genes for co-expression in P. pastoris | This study |
| pL3Z-ARO47TAL | pL3Z-His-derived vector assembled via Golden Gate cloning, harboring scARO4 (K229L), scARO7 (G141S), and haTAL genes (the tri-gene cassette for p-CA biosynthesis) | This study |

**Table S3. Sequences and applications of primers used in this study.**

| **Primer Name** | **Nucleotide Sequence (5′→3′)** | **Application** |
| --- | --- | --- |
| PpGAPDH-dF | cgagggtaagctcaagggtg | qPCR |
| PpGAPDH-dR | ttgaataccggcagaagcgt |  |
| ARO4-F | atcccagccgaaggtaaagc |  |
| ARO4-R | gacagcagcagccaatttcc |  |
| ARO7-F | gtgtggaccctaccaacgag |  |
| ARO7-R | gcaagtattccacctcaacttcc |  |
| TAL-F | atctgcctctttggttggca |  |
| TAL-R | ctcagcgtgcgttggaaatc |  |
| 5′AOX1 | gactggttccaattgacaagc | Strain construction and verification |
| 3′AOX1 | gcaaatggcattctgacatcc |  |
| scARO4-F | gagtgaatctccaatgttcgc |  |
| scARO4-R | tgtctgacagcagcagcc |  |
| scARO7-F | gagatgaattagttagaatggaggattc |  |
| scARO7-R | ccttcttagcaagtattccacctc |  |
| haTAL-F | ctaacaggtgcaggtctaacc |  |
| haTAL-R | ctatctaaggtttcttgaattgcctg |  |
| GGA-CASS1-F | taacggtctcccagaaacatccaaagacgaaag |  |
| GGA-CASS1-R | attgggtctcccactctcacttaatcttctgtact |  |
| GGA-CASS2-F | taacggtctccagtgaacatccaaagacgaaag |  |
| GGA-CASS2-R | aggtctcccctgctcacttaatcttctgtact |  |
| GGA-CASS3-F | taacggtctcccaggaacatccaaagacgaaag |  |
| GGA-CASS3-R | aggtctcctcctctcacttaatcttctgtact |  |
| Gibson-5AOX-F | gatcaaaaaacaactaattattcgaaa |  |
| Gibson-3AOX-F | aacagtcatgtctaaggctacaaac |  |
| Gibson-pMPIC-F | tcccccttttcctttgtc |  |
| Gibson-pMPIC-R | ggtttagttcctcaccttg |  |
| Gibson-gene-F | gatctaacatccaaagacg |  |
| Gibson-gene-R | tctcacttaatcttctgtactc |  |
| int1-up-F | gagttgcaagtggacgagga |  |
| int1-up-R | cgttagcatttcaacgaacc |  |
| int1-dn-F | tggcgctggcaaattgttgc |  |
| int1-dn-R | caatatcgttagctggttgc |  |
| SgRNA-int1 | cccagtaaatacttcagata |  |

**Abbreviations and their full names**

| **Abbreviations** | **full names** |
| --- | --- |
| *P. pastoris* | *Pichia pastoris (Komagataella phaffii)* |
| *E. coli* | *Escherichia coli* |
| Δ*Ku70* | *Ku70* gene deletion |
| *ARO4*ᶠᵇʳ | Feedback inhibition-resistant *ARO4* (3-deoxy-D-arabino-heptulosonate-7-phosphate synthase) |
| *ARO7*ᶠᵇʳ | Feedback inhibition-resistant *ARO7* (chorismate mutase) |
| *scARO4* | *ARO4* from Saccharomyces cerevisiae |
| *scARO7* | *ARO7* from Saccharomyces cerevisiae |
| ***haTAL*** | Tyrosine ammonia-lyase from Herpetosiphon aurantiacus |
| ***GAPDH*** | **Glyceraldehyde-3-phosphate dehydrogenase** |
| *HIS4* | Histidine 4 gene |
| BMMY | Buffered Methanol-complex Medium |
| BSM | Basal Salt Medium |
| LB | Luria-Bertani Medium |
| YE | Yeast extract |
| YNB | Yeast Nitrogen Base (without amino acids) |
| YPD | Yeast Extract Peptone Dextrose Medium |
| XuMP | Xylulose monophosphate pathway |
| p-CA | *p*-Coumaric acid |
| DAHP | 3-Deoxy-D-arabino-heptulosonate-7-phosphate |
| DHAP | Dihydroxyacetone phosphate |
| E4P | Erythrose-4-phosphate |
| F6P | Fructose-6-phosphate |
| FBP | Fructose-1,6-bisphosphate |
| GAP | Glyceraldehyde-3-phosphate |
| PEP | Phosphoenolpyruvate |
| Ru5P | Ribulose-5-phosphate |
| Xu5P | Xylulose-5-phosphate |
| qPCR | Quantitative real-time PCR |
| Cas9 | CRISPR-associated protein 9 |
| CRISPR | Clustered Regularly Interspaced Short Palindromic Repeats |
| s.d. | Standard deviation |
| HPLC | High-performance liquid chromatography |
| OD_600_ | Optical density at 600 nm |
| Zeocin | Zeocin resistance |
| **WGS** | Whole-genome sequencing |
